# Autophagy-Inducing Factor Atg1 Is Required for Virulence in the Pathogenic Fungus *Candida glabrata*

**DOI:** 10.3389/fmicb.2019.00027

**Published:** 2019-01-25

**Authors:** Shintaro Shimamura, Taiga Miyazaki, Masato Tashiro, Takahiro Takazono, Tomomi Saijo, Kazuko Yamamoto, Yoshifumi Imamura, Koichi Izumikawa, Katsunori Yanagihara, Shigeru Kohno, Hiroshi Mukae

**Affiliations:** ^1^Department of Respiratory Medicine, Nagasaki University Hospital, Nagasaki, Japan; ^2^Department of Infectious Diseases, Nagasaki University Graduate School of Biomedical Sciences, Nagasaki, Japan; ^3^Department of Laboratory Medicine, Nagasaki University Graduate School of Biomedical Sciences, Nagasaki, Japan

**Keywords:** autophagy, Atg1, *Candida glabrata*, virulence, reactive oxygen species

## Abstract

*Candida glabrata* is one of the leading causes of candidiasis and serious invasive infections in hosts with weakened immune systems. *C. glabrata* is a haploid budding yeast that resides in healthy hosts. Little is known about the mechanisms of *C. glabrata* virulence. Autophagy is a ‘self-eating’ process developed in eukaryotes to recycle molecules for adaptation to various environments. Autophagy is speculated to play a role in pathogen virulence by supplying sources of essential proteins for survival in severe host environments. Here, we investigated the effects of defective autophagy on *C. glabrata* virulence. Autophagy was induced by nitrogen starvation and hydrogen peroxide (H_2_O_2_) in *C. glabrata*. A mutant strain lacking CgAtg1, an autophagy-inducing factor, was generated and confirmed to be deficient for autophagy. The *Cgatg1*Δ strain was sensitive to nitrogen starvation and H_2_O_2_, died rapidly in water without any nutrients, and showed high intracellular ROS levels compared with the wild-type strain and the *CgATG1*-reconstituted strain *in vitro*. Upon infecting mouse peritoneal macrophages, the *Cgatg1*Δ strain showed higher mortality from phagocytosis by macrophages. Finally, *in vivo* experiments were performed using two mouse models of disseminated candidiasis and intra-abdominal candidiasis. The *Cgatg1*Δ strain showed significantly decreased CFUs in the organs of the two mouse models. These results suggest that autophagy contributes to *C. glabrata* virulence by conferring resistance to unstable nutrient environments and immune defense of hosts, and that Atg1 is a novel fitness factor in *Candida* species.

## Graphical Abstract

The deletion of *ATG1* disrupted autophagy function in *C. glabrata*, leading to increased sensitivity to hydrogen peroxide (H_2_O_2_) and nutrient starvation. These phenotypes were associated with decreased fitness and virulences in host environments.

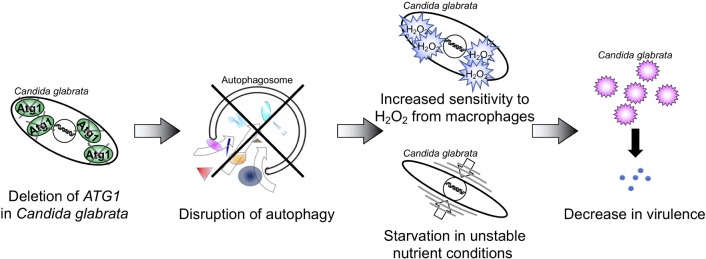

## Introduction

Autophagy is an evolutionarily conserved biological process in eukaryotes that involves the degradation of cytosolic molecules for recycling intracellular materials (Klionsky et al., 2016). Autophagy has several functions: supplying nutrients under starvation conditions, maintaining homeostasis and protein levels, clearing damaged and/or non-functional proteins, and preventing bacterial and viral infections. These functions contribute to cell longevity, development, and differentiation as well as tumor suppression (Jin et al., 2017; Chen et al., 2018; Hansen et al., 2018). In the induction of autophagy, autophagosomes, which are double-membrane cytosolic vesicles, are formed to incorporate target proteins. In the model yeast *Saccharomyces cerevisiae*, autophagosomes fuse with vacuoles, and proteins are degraded in the vacuoles.

Fungal pathogens also use autophagy to survive in a host environment with unstable nutrient supply (Khan et al., 2012). However, little is known about the contribution of fungal autophagy to their persistence and virulence in hosts. In addition, immune defenses such as macrophages generate ROS to kill infecting pathogens (Gonzalez-Parraga et al., 2003; Thorpe et al., 2004), but it has remained obscure whether fungal autophagy affects this oxidative stress response.

*Candida* species are a genus of opportunistic fungal pathogens that cause severe invasive infections in immunocompromised patients (Miceli et al., 2011). *Candida glabrata* is the second most common cause of candidiasis in humans (Roetzer et al., 2011). The genetic background of *C. glabrata* is closely related to that of *S. cerevisiae*. *C. glabrata* is a commensal yeast and capable of surviving in the host longer than other *Candida* species (Roetzer et al., 2011). We hypothesized that autophagy contributes to these functions in *C. glabrata*.

Autophagy has several subcategories. Macroautophagy covers a broad range of protein degradation processes that are mainly induced upon starvation (Suzuki, 2013). Pexophagy and mitophagy are specific to the degradation of damaged peroxisomes and mitochondria, respectively (Suzuki, 2013). Recently, pexophagy and mitophagy have been suggested to be related to *C. glabrata* virulence (Roetzer et al., 2010; Nagi et al., 2016). In the present study, we analyzed macroautophagy.

Macroautophagy (hereinafter simply referred to as autophagy) is induced by Atg proteins in yeasts (Yorimitsu and Klionsky, 2005). Atg1 is a component of an Atg protein complex and is essential for autophagy induction (Wang and Kundu, 2017). *Candida glabrata* Atg1 (CgAtg1) is also predicted to be important for autophagy, because ATG genes are highly conserved between *S. cerevisiae* and *C. glabrata*. Here, we showed that *C. glabrata* autophagy is induced by nitrogen starvation and H_2_O_2_. The *CgATG1*-deleted mutant of *C. glabrata* exhibited deficient adaptation to starvation and H_2_O_2_ *in vitro*. An *ex vivo* experiment using mouse peritoneal macrophages demonstrated that the *Cgatg1*Δ strain was phagocytosed by macrophages and showed low viability. Autophagy was revealed to be important for *C. glabrata* survival in two mouse models of invasive candidiasis.

## Materials and Methods

### Ethics Statement

Animal experiments were conducted according to the Guide for the Care and Use of Laboratory Animals (National Research Council, National Academy Press, Washington, DC, 2011) and all of the institutional regulations and guidelines for animal experimentation after pertinent review and approval by the Institutional Animal Care and Use Committee of Nagasaki University (approval number 1407281164-4).

### Culture Conditions

*C. glabrata* was routinely cultured at 30°C in SC-trp (Dunham et al., 2015) or YPD agar [1% yeast extract, 2% peptone, 2% dextrose, and 2% Bacto agar (BD Biosciences, B242720)], unless otherwise indicated. SD-N [0.17% yeast nitrogen base without amino acids and ammonium sulfate (BD Biosciences, 233520) and 2% dextrose] was used for the nitrogen starvation condition (Budovskaya et al., 2004).

### Strain and Plasmid Construction

*C. glabrata* strains, plasmids, and primers used in this study are listed in Tables 1–3, respectively. Sequence information of *C. glabrata* genes was obtained from the *Candida* genome database^1^.

**Table 1:** *C. glabrata* strains used in this study.

Strain	Genotype or description	Reference
CBS138	*Candida glabrata* wild-type (ATCC2001)	Dujon et al., 2004
2001T	CBS138/*trp1*Δ	Kitada et al., 1995
wild-type	2001T containing pCgACT	Miyazaki et al., 2011
WT+*GFP-CgATG8*	2001T containing pCgACT-GFP-CgATG8	This study
WT+*CgCTA1*-OE	2001T containing pCgACTP-CgCTA1	Nishikawa et al., 2016
KUE200	CBS138/*his3Δ, trp1Δ, FRT-YKU80*	Ueno et al., 2007
*Cgatg1*Δ	KUE200/*atg1Δ::HIS3* containing pCgACT	This study
*Cgatg1*Δ+*CgATG1*	KUE200/*atg1Δ::HIS3* containing pCgACT-CgATG1	This study
*Cgatg1*Δ+*GFP-CgATG8*	KUE200/*atg1Δ::HIS3* containing pCgACT-GFP-CgATG8	This study
*Cgatg1*Δ+*CgCTA1*-OE	KUE200/*atg1Δ::HIS3* containing pCgACTP-CgCTA1	This study


**Table 2:** Plasmids used in this study.

Plasmid	Description	Reference
pBSK-HIS	pBluescript II SK+ containing *C. glabrata HIS3* at the *Xho* I site	Miyazaki et al., 2010a
pCgACT	*C. glabrata* centromere-based plasmid containing autonomously replicating sequence and *C. glabrata TRP1*	Kitada et al., 1996
pCgACT-CgATG1	*C. glabrata ATG1* promoter, ORF, and 3′-UTR were inserted into the *Bam*H I-*Sal* I site of pCgACT.	This study
pCgACT-GFP-CgATG8	*C. glabrata ATG8* promoter, N-terminally GFP-tagged ORF, and 3′-UTR were inserted into the *Bam*H I-*Sal* I site of pCgACT.	This study
pCgACTP	*S. cerevisiae PGK1* promoter and *C. glabrata HIS3* 3′UTR were inserted into the *Sac I-Kpn I* site of pCgACT.	Miyazaki et al., 2010a
pCgACTP-CgCTA1	*C. glabrata CTA1* ORF was inserted into the *Bam*H I-*Sal* I site of pCgACTP.	Nishikawa et al., 2016


**Table 3:** Primers used in this study.

Gene deletion	Sequence (5′ to 3′)
CgATG1-100F	GTTATCCAAAAGCAATATAGCATAAAGTTCACAATTTGAATTATAACGGATTTGCTAGTTAGGTCTTAAAAATTAGTACTCGAGATGAGCTCCC
	AAAAGTAATACGACTCACTATAGGGC
CgATG1-100R	AGATCAAAAAAATTGACAACTAGTAACTATTATCAGAGAATAAGTCTATTTTTAGGTTATTGTAAAACAACCAATTAATGCATCCCTTTTGGATG
	AATCCGCTCTAGAACTAGTGGATCC
**Plasmid construction**	**Sequence (5′ to 3′)**
CgATG1-F(-596FL)-Sal	AAAGTCGACCCATCAGGTTAGCAGGTGTC
CgATG1-R(+356FL)-Kpn	AAGGTACCGTCATCAAGTGGTCGTAGGC
CgATG8-up500F	GACGGCCAGTGAATTCTTCCATGAAATCATTTCCTG
CgATG8-down771R	ATGCCTGCAGGTCGACATAGAGGAGATGGTGGAGTAGC
GFP-F	AATACCACTCCCGGGAAAATGTCTAAAGGTGAAGAATTATTC
GFP-R	GAATGATGACTTCATCCATGGTTTGTACAATTCATCCATACCATG
CgATG8-F	ATGAAGTCATCATTCAAAAGTG
CgATG8-upR	CCCGGGAGTGGTATTGAATTTCTTGG


The *C. glabrata atg1*Δ strain was constructed using a one-step PCR-based technique as described previously (Miyazaki et al., 2010a, 2011). Briefly, an *CgATG1* deletion construct was amplified from pBSK-HIS using primers tagged with 100-bp sequences homologous to the flanking regions of the *CgATG1* ORF (CgATG1-100F and CgATG1-100R). *C. glabrata* parent strains were transformed with the deletion construct, and the resulting transformants were selected by histidine prototrophy (Miyazaki et al., 2011). Successful homologous recombination was verified by diagnostic PCR, and the absence of *CgATG1* mRNA expression was confirmed by real-time qRT-PCR (data not shown). Transformation of *C. glabrata* was performed using the lithium acetate protocol, as described previously (Cormack and Falkow, 1999).

pCgACT-CgATG1, in which *CgATG1* was expressed under the control of the *CgATG1* native promoter, was constructed as follows: a 3,781-bp DNA fragment containing the *CgATG1* promoter, ORF, and 3′-UTR was amplified using CgATG1-F(-596FL)-Sal and CgATG1-R(+356FL)-Kpn, digested with SalI and KpnI, and inserted into the SalI-KpnI site of pCgACT (Kitada et al., 1996). An *CgATG1*-reconstituted strain and its control strains were constructed by transformation of the *Cgatg1*Δ strain with pCgACT-CgATG1 and pCgACT, respectively. They were selected by tryptophan prototrophy and verified by qRT-PCR (data not shown).

pCgACT-GFP-CgATG8, in which N-terminally GFP-tagged *CgATG8* was expressed under the control of the *CgATG8* native promoter, was constructed using In-Fusion HD Cloning Plus CE (Clontech Laboratories, 638916). Briefly, a 1,600-bp DNA fragment containing the *CgATG8* promoter, ORF, and 3′UTR was amplified using CgATG8-up500F and CgATG8-down771R, and inserted into the EcoRI-SalI site of pCgACT by the In-Fusion reaction to generate pCgACT-CgATG8. GFP (yEGFP1) was amplified from pYGFP1 (Cormack et al., 1997) using GFP-F and GFP-R, and inserted between the *CgATG8* promoter and the ORF in pCgACT-CgATG8 by the In-Fusion reaction to generate pCgACT-GFP-CgATG8. The insertion site of the vector was produced by a PCR reaction using pCgACT-CgATG8 as the template and the primers CgATG8-F and CgATG8-upR. The *C. glabrata* wild-type strain 2001T and the *Cgatg1*Δ strain were transformed with pCgACT-GFP-CgATG8.

### Growth Curve Construction

Logarithmic-phase *C. glabrata* cells were adjusted to 5 × 10^6^ cells/ml and incubated in SC-trp broth at 37°C. The number of cells was counted at 2, 4, 6, 8, 24, and 48 h. Doubling times were calculated as previously described (Geber et al., 1995), except that the cells were counted using a hemocytometer instead of OD_600_. The averages of the doubling times were obtained from four independent experiments.

### Spot Assay

Spot assays were performed as described previously (Miyazaki et al., 2010b). Briefly, logarithmic-phase cells grown in SC-trp broth were harvested and adjusted to a concentration of 2 × 10^7^ cells/ml. Serial 10-fold dilutions were prepared, and 5 μl of each dilution was spotted onto agar plates and incubated at 30°C for 48 h, unless otherwise indicated.

### Immunoblotting

Anti-GFP (Roche, 11814460001), anti-Pgk1 (OriGene EU, AP21371AF-N), anti-mouse IgG-hrp (GE Healthcare, NA931V), and anti-rabbit IgG-hrp (GE Healthcare, NA934V) antibodies were purchased. Logarithmic-phase cells were harvested and lysed using a Minute Total Protein Extraction Kit for Microbes with Thick Cell Walls (Invent Biotechnologies, YT-015) according to the accompanying instructions. Lysates were separated by SDS–PAGE and transferred to a polyvinylidene difluoride membrane (BIO-RAD, 1704156). Each protein was detected using the indicated antibodies, enhanced chemiluminescent substrate (Thermo Fisher Scientific, 34096), and a ChemiDoc Touch Imaging System (BIO-RAD, Hercules, CA, United States).

### Measurement of Intracellular ROS Levels

Intracellular ROS levels in *C. glabrata* were analyzed by measuring fluorescent DCF derived from the reaction of CM-H_2_DCFDA (Thermo Fisher Scientific, C6827) and ROS. Cell-permeable CM-H_2_DCFDA is not fluorescent; it reacts with ROS to produce fluorescent DCF. Logarithmic-phase cells were washed with PBS, resuspended in PBS with 10 μM CM-H_2_DCFDA, and incubated at 30°C for 1 h. The cells were washed, resuspended in SC-trp with H_2_O_2_ (Wako, 084-07441), *tert*-butyl hydroperoxide (Wako, 026-13451), menadione (Wako, 132-08132), or diamide (Sigma-Aldrich, D3648), and cultured at 30°C for 1 h. The cells were washed and resuspended in PBS, and fluorescence was measured with a PHERAstar FS multi-mode microplate reader (BMG LABTECH, Offenburg, Germany) at 485 nm fluorescence excitation wavelength and 520 nm emission wavelength. The cell count of each sample was determined, and relative fluorescence intensity per cell was calculated.

### Lifespan Assay

Incubation of 2.5 × 10^6^/ml cells was started in ultrapure water (Thermo Fisher Scientific, 10977-023) at 30°C. The cells were harvested at multiple time points and plated on YPD agar. The number of viable cells was determined by counting colonies after incubation for 36 h at 30°C, and the number of CFUs per ml was calculated.

### Macrophage Infection Assay

To collect mouse peritoneal macrophages, peritoneal exudates were obtained from 8-week-old male BALB/c mice (Japan SLC, Shizuoka, Japan) by lavage 4 days after intraperitoneal injection of 2 ml of sterile 4% thioglycollate broth (BD Biosciences, 211716). After washing with DMEM, peritoneal macrophages were incubated in DMEM with 10% FBS and 100 μg/ml gentamycin (Nacalai Tesque, 11980-14) at 37°C under 5% CO_2_. Macrophages of 1.5 × 10^5^ cells were seeded in 24-well plates and incubated in DMEM with 10% FBS for 24 h. For infection assays, 2.5 × 10^5^ *C. glabrata* cells were resuspended in DMEM, added to the macrophages, and incubated. The actual *C. glabrata* cell number was confirmed by plating the cell suspension on YPD agar. Two hours after infection, the infected macrophages were washed thrice with PBS to remove non-phagocytosed *C. glabrata* cells and incubated in fresh DMEM. At multiple time points post-infection, the infected macrophages were observed under a microscope (BZ-X700, Keyence, Osaka, Japan). Then, the macrophages were lysed in water, and phagocytosed *C. glabrata* cells were plated on YPD agar containing streptomycin sulfate salt (Sigma-Aldrich, S1277) and penicillin G sodium salt (Sigma-Aldrich, P3032). The number of viable *C. glabrata* cells was determined by counting colonies after incubation for 36 h at 30°C, and the number of CFU per well was calculated.

### Mouse Studies

Mouse experiments were performed as described previously (Miyazaki et al., 2010a,b). Briefly, to prepare cells for injection, cultured *C. glabrata* cells were resuspended in sterile saline and adjusted to 5 × 10^8^ or 1 × 10^9^ cells/ml. The actual *C. glabrata* concentration was confirmed by plating the adjusted cell suspension on YPD agar. The tail veins or abdominal cavities of 8-week-old female BALB/c mice (Japan SLC) were injected with 200 μl suspensions of each *C. glabrata* strain. The mice were sacrificed 7 days post-injection, and the target organs were excised. The organs were homogenized, diluted, and plated on YPD agar containing streptomycin sulfate salt and penicillin G sodium salt. Colonies were counted after incubation for 36 h at 30°C, and the number of CFU per organ was calculated.

## Results

### Atg1 Is Necessary for Normal Growth Rate, Survival, and Autophagy During Nitrogen Starvation and Oxidative Stress

**FIGURE 1:**
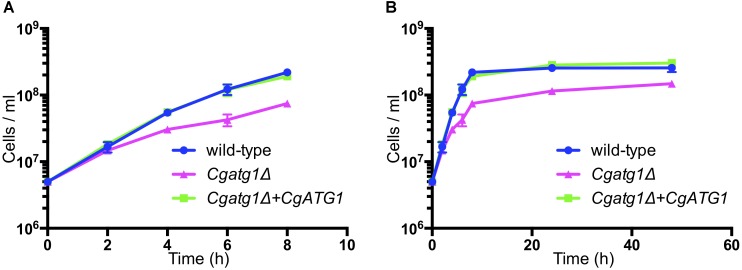
Growth curves of *C. glabrata* strains. Logarithmic-phase *C. glabrata* cells were grown in SC-trp broth at 37°C and counted at multiple time points. Graphs show data for the first 8 h of culture **(A)** to focus on the log phase and 48 h of culture **(B)**. *C. glabrata* strains: wild-type (*blue circles*), *Cgatg1*Δ (*magenta triangles*), and *Cgatg1*Δ+*CgATG1* (*CgATG1*-reconstituted *Cgatg1*Δ, *green squares*). The means ± SE of four independent experiments are shown. Doubling times: wild-type, 1.173 h; *Cgatg1*Δ, 1.912 h; *Cgatg1*Δ+*CgATG1*, 1.254 h.

**FIGURE 2:**
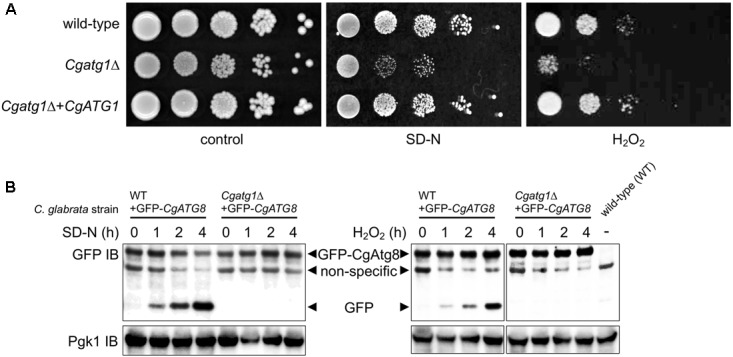
*CgATG1*-deleted *C. glabrata* is sensitive to nitrogen starvation and H_2_O_2_. **(A)** Logarithmic-phase cells were serially 10-fold diluted, spotted onto agar plates of SC-trp, SD-N, and SC-trp with 13.3 mM H_2_O_2_, and cultured at 30°C for 48 h. **(B)** N-terminally GFP-tagged CgAtg8 was expressed in the wild-type strain and the *Cgatg1*Δ strain. Cells were cultured in SD-N (*left*) or SC-trp with 13.3 mM H_2_O_2_ (*right*) for the indicated hours. Protein was extracted and immunoblotted using anti-GFP antibody and anti-Pgk1 antibody as a loading control. The protein extract of the wild-type strain without GFP-Atg8 expression was loaded as a negative control.

The *Cgatg1*Δ strain showed slightly slower growth than the wild-type strain and the *CgATG1*-reconstituted strain under the SC-trp growth condition (Figure 1). Doubling times of the wild-type, *Cgatg1*Δ, and *CgATG1*-reconstituted strains in SC-trp medium were 1.173, 1.912, and 1.254 h, respectively. Spot assays revealed growth defects in the *Cgatg1*Δ strain under the nitrogen starvation condition and in the presence of H_2_O_2_ (Figure 2A). These phenotypes recovered to the wild-type levels by the reintroduction of intact *CgATG1* into the mutant.

It remains to be established whether or not autophagy is induced by H_2_O_2_ in *C. glabrata*. The induction of autophagy by starvation and the necessity of CgAtg1 for autophagy also need to be confirmed, as reports of autophagy in *C. glabrata* are still few. In *S. cerevisiae*, GFP-Atg8 is known to be cleaved by autophagy, resulting in the release of GFP, which can be detected as a marker of autophagy (Shintani and Klionsky, 2004). GFP-CgAtg8 was expressed in *C. glabrata* from a transformed plasmid containing the *CgATG8* native promoter followed by an N-terminally GFP-tagged *CgATG8* ORF. GFP-Atg8 and GFP bands were specifically observed in GFP-Atg8-expressing strains, but not in the negative control strain containing an empty vector (Figure 2B). GFP bands gradually became more intense as a result of nitrogen starvation and H_2_O_2_ exposure in the wild-type strain, whereas no GFP bands were observed over the time-course in the *Cgatg1*Δ strain (Figure 2B), indicating that autophagy was induced in response to nitrogen starvation and H_2_O_2_ exposure in a CgAtg1-dependent manner in *C. glabrata*.

**FIGURE 3:**
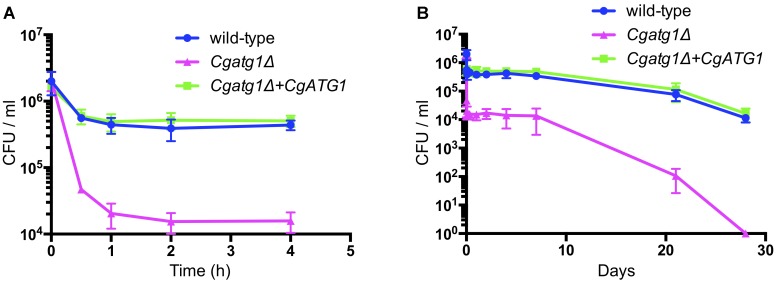
CgAtg1 is important for *C. glabrata* viability under nutrient starvation conditions. *C. glabrata* strains were incubated in pure water at 30°C, harvested at multiple time points, and cultured in YPD agar. Then, the number of CFU was counted. Graphs show data for the first 4 h **(A)** and over 28 days **(B)** of incubation. The means ± SE of three independent experiments are shown.

In addition to the spot assay, a lifespan assay was performed to examine the sensitivity of the *Cgatg1*Δ strain to the starvation condition using pure water. Compared to the wild-type strain, the *Cgatg1*Δ strain showed a rapid decline in viability within 2 h (Figure 3A). Changes in *C. glabrata* viability became gradual after 2 h, and the *Cgatg1*Δ cells died after 4 weeks, in contrast to the wild-type cells that remained viable at 4 weeks in water (Figure 3B). Overall, these results suggest that autophagy plays a role in the adaptation to starvation in *C. glabrata*.

### Analyses of the Correlation Between H_2_O_2_ Sensitivity and Intracellular ROS Levels

**FIGURE 4:**
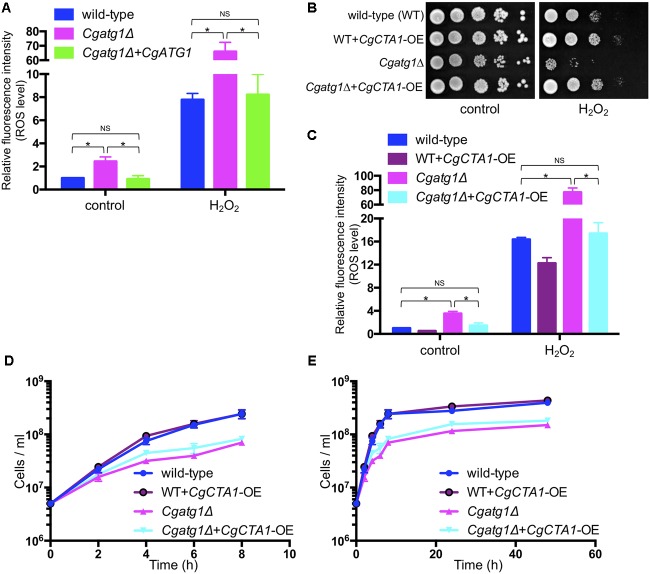
Analyses of intracellular ROS level with H_2_O_2_ and effect of catalase. **(A)** *C. glabrata* strains containing CM-H_2_DCFDA were cultured in SC-trp with/without 13.3 mM H_2_O_2_ at 30°C for 1 h, and the fluorescence produced by the reaction of CM-H_2_DCFDA and ROS in the cells was measured. Relative fluorescence intensity per cell was calculated, and the fluorescence intensity of the wild-type strain without H_2_O_2_ was defined as 1. The means ± SE of three independent experiments are shown. Statistical analyses were performed using two-tailed Student’s *t-*tests. ^∗^*P* < 0.01; NS, no significance (*P* > 0.05). **(B–E)** Spot assays **(B)**, measurement of intracellular ROS levels **(C)**, and growth curve construction **(D,E)** were performed as in Figure 2A **(B)**, Figure 4A **(C)**, and Figures 1A,B **(D,E)** to analyze the effect of *CgCTA1* overexpression. *C. glabrata* strains: wild-type (*blue*), WT+*CgCTA1*-OE (*purple*), *Cgatg1*Δ (*magenta*), and *Cgatg1*Δ+*CgCTA1*-OE (*cyan*). **(D,E)** Doubling times: wild-type, 1.136 h; WT+*CgCTA1*-OE, 1.032 h; *Cgatg1*Δ, 1.995 h; *Cgatg1*Δ+*CgCTA1*-OE, 1.499 h.

Intracellular ROS levels were increased by H_2_O_2_ in all the strains tested (Figure 4A). The *Cgatg1*Δ strain had a higher ROS level than the wild-type strain and the *CgATG1*-reconstituted strain regardless of the presence or absence of H_2_O_2_. High ROS levels in the *Cgatg1*Δ strain were also observed after exposure to other ROS-generating agents, including *tert*-butyl hydroperoxide (an oxidant), menadione (a superoxide-generating agent), and diamide (an inhibitor of hydroxyl radical metabolism by decreasing the glutathione pool) (Supplementary Figure S1). The elevations of ROS levels in the *Cgatg1*Δ strain by these three ROS-generating agents were mild (approximately 10- to 15-fold relative to the control), while H_2_O_2_ markedly elevated the ROS level in the *Cgatg1*Δ strain (over 60-fold relative to the control). The sensitivity of the *Cgatg1*Δ strain to H_2_O_2_ was eliminated by the overexpression of *CgCTA1* (*CaTalase A1*) that encodes a catalase involved in the metabolism of H_2_O_2_ (Figure 4B). Consistent with the spot assay, the intracellular ROS amount in the *Cgatg1*Δ strain decreased to the level of the wild-type strain by *CgCTA1* overexpression (Figure 4C). These results suggest that autophagy affects intracellular ROS levels and resistance to H_2_O_2_ in *C. glabrata*. *CgCTA1* overexpression partially rescued the growth rate of the *Cgatg1*Δ strain under the SC-trp growth condition (Figures 4D,E). Doubling times of the wild-type, *CgCTA1*-overexpressed wild-type, *Cgatg1*Δ, and *CgCTA1*-overexpressed *Cgatg1*Δ strains in SC-trp medium were 1.136, 1.032, 1.995, and 1.499 h, respectively.

### *CgATG1* Deletion Decreases Resistance of *C. glabrata* to Macrophages

**FIGURE 5:**
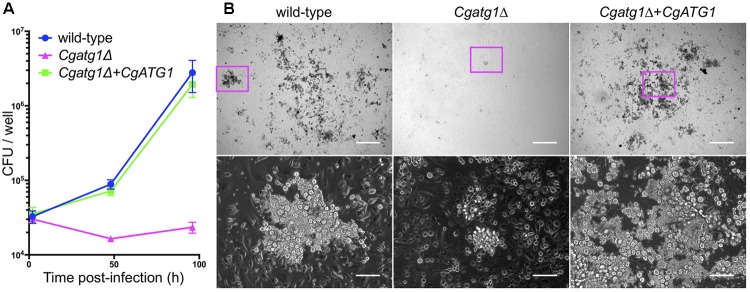
*CgATG1*-deleted *C. glabrata* is sensitive to phagocytosis by macrophages. Mouse peritoneal macrophages were infected with *C. glabrata* strains in DMEM supplemented with 10% FBS. **(A)** Macrophages including *C. glabrata* were harvested at 2, 48, and 96 h post-infection, lysed in water, and plated on YPD agar for CFU count. The means ± SE of three independent experiments are shown. **(B)** Microscopic images of macrophages infected with *C. glabrata* for 96 h. Bottom panels are enlarged images of magenta boxes in the respective upper panels. Growing *C. glabrata* cells were observed in the wild-type strain and the *Cgatg1*Δ+*CgATG1* strain, but rarely in the *Cgatg1*Δ strain. Bar: 500 μm (upper), 100 μm (bottom).

Activated macrophages generate H_2_O_2_ for phagocytosis (Forman and Torres, 2001). In addition, nutrients are restricted in the engulfed state by macrophages. Autophagy in *C. glabrata* might act to resist the immune response. To assess this hypothesis, an *ex vivo* experiment was performed using macrophages collected from mouse abdominal cavities. *C. glabrata* strains were added to wells containing the peritoneal macrophages and co-incubated, and viable *C. glabrata* cells phagocytosed by the macrophages were counted. Growth of the *Cgatg1*Δ strain was suppressed during the 96-h co-incubation, whereas growth of the wild-type strain and the *CgATG1*-reconstituted strain was increased despite phagocytosis by macrophages (Figure 5A). During the 96-h period post-infection, the increase in the number of wild-type *C. glabrata* cells was remarkable, whereas *Cgatg1*Δ cells were hardly observed (Figure 5B). This suggests that autophagy is required for resistance to macrophages in *C. glabrata*.

### CgAtg1 Is Important for *C. glabrata* Persistence in Hosts

**FIGURE 6:**
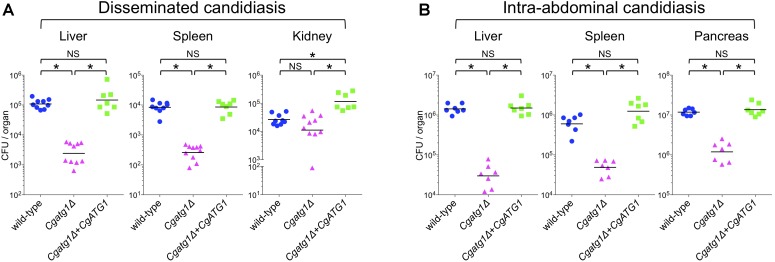
*CgATG1* deletion decreases *C. glabrata* viability in mouse models of disseminated and intra-abdominal candidiasis. Groups of 7 to 10 BALB/c mice were inoculated with 1 × 10^8^ cells intravenously **(A)** or 2 × 10^8^ cells intraperitoneally **(B)** of each *C. glabrata* strain. Target organs were excised 7 days after injection. The organ homogenates were plated on YPD agar for CFU count. Numbers of CFU from each organ are indicated for individual mice in the scatter plots. Each geometric mean is shown by a bar. Statistical analyses were performed using the Wilcoxon Mann–Whitney test. ^∗^*P* < 0.01; NS, no significance (*P* > 0.05). Representative data of two independent experiments are shown.

In mouse experiments with *C. glabrata*, DC mouse models are used because *C. glabrata* frequently spreads in patients through blood flow. Macrophages mainly cause inflammation in the abdominal cavity, although they also function in the blood and subsequent organs. To observe *C. glabrata* intraperitoneal infection, a mouse model of IAC was also employed. Liver, spleen, and kidney are the typical organs examined in the DC mouse model. The pancreas is the predominant organ for *C. glabrata* infection in the IAC mouse model, while the kidney is retroperitoneal and has not yielded stable results in previous studies (Cheng et al., 2013, 2014) or in our preliminary experiments (data not shown). Decreases in the number of CFU were observed in the liver and spleen of the DC mouse model (Figure 6A) and all organs examined in the IAC mouse model (Figure 6B) when the mice were colonized by the *Cgatg1*Δ strain. In the kidney of the DC mouse model, the number of CFU slightly decreased in the *Cgatg1*Δ strain without statistical significance (Figure 6A). These results suggest that autophagy positively affects *C. glabrata* viability in the host.

## Discussion

Research on autophagy as a driving force of virulence is still in its developing stages compared to fundamental studies in humans and *S. cerevisiae* (Klionsky et al., 2016). We examined autophagy in the pathogenic fungus *C. glabrata* by conducting *in vitro, ex vivo*, and *in vivo* experiments, and found that autophagy has positive effects on *C. glabrata* virulence.

*In vitro*, CgAtg1 was required for autophagy, and the *Cgatg1*Δ strain was sensitive to the nutrient starvation condition, suggesting that autophagy in *C. glabrata* is important for adaptation to starvation, which has been observed in other fungal species such as *Candida albicans* and *Cryptococcus neoformans* (Palmer et al., 2008; Yu et al., 2015). Furthermore, rapid cell death and long-term survival defects were noted in the *Cgatg1*Δ strain in the lifespan assay, suggesting that autophagy functions in both adaptation to sudden environmental changes and for persistence of *C. glabrata* in hosts.

In pathogenic fungi, autophagy has been indicated to be dispensable for *C. albicans* virulence, whereas it is required for the infection process of *C. neoformans* (Palmer et al., 2008). *C. glabrata* is also a commensal yeast, but its infection mechanism is different from that of *C. albicans*. *C. albicans* forms hyphae to invade across the host environment and to escape from nutrient-starved loci and macrophages (Desai, 2018). *C. glabrata* seems to require autophagy because it lacks the capacity to form hyphae but adheres to many different objects for a long time (Timmermans et al., 2018).

The functional mechanisms of autophagy in oxidative stress response remain to be elucidated. This study showed high intracellular ROS levels in the *Cgatg1*Δ strain, especially by H_2_O_2_ addition. *CgCTA1* overexpression rescued the resistance of the *Cgatg1*Δ strain to H_2_O_2_; however, this result does not indicate that autophagy promotes the expression of *CgCTA1*. Rather, factors other than the expression level of *CgCTA1* are thought to be the cause of the high ROS levels in the *Cgatg1*Δ strain, as ROS elevation was also induced by other ROS-generating agents that are not metabolized by Cta1 (Supplementary Figure S1). Indeed, the rescue of the growth rate of the *Cgatg1*Δ strain in SC-trp by *CTA1* overexpression was only partial and not up to the level of the wild-type strain (Figures 4D,E). Cta1 is considered to reverse the defect of the *Cgatg1*Δ strain only for adaptation to H_2_O_2_, but not for the overall functions of autophagy. Multiple factors are involved in the ROS elevation, as intracellular ROS levels including H_2_O_2_ are changed by various stresses and regulatory factors, and autophagy affects a wide range of molecules.

In addition to the regulation of intracellular ROS, it is speculated that fungal autophagy might affect the repair of cellular damage caused by H_2_O_2_. The adaptation to H_2_O_2_ has been analyzed in pexophagy in mammals and plants (Lee et al., 2014; Zientara-Rytter and Subramani, 2016). Peroxisomes generate H_2_O_2_ and are self-oxidized. These damaged peroxisomes are degraded by pexophagy for recycling, which leads to efficient maintenance of functional and fresh peroxisomes and the cleaning of junk; this is called ‘quality control.’ Recent studies in mammals have suggested that macroautophagy is also activated by H_2_O_2_ (Filomeni et al., 2015; Yin et al., 2015; Nah et al., 2017). From previous reports and this study, autophagy is supposed to have a two-step function in the adaptation to H_2_O_2_ (Supplementary Figure S2). The first step is the metabolism of overall ROS, and the second is the quality control of proteins and organelles injured by H_2_O_2_. Further investigations are needed to elucidate the precise molecular mechanisms in *C. glabrata* and other species.

The *Cgatg1*Δ strain exhibited slightly impaired growth and high ROS levels in the SC-trp medium (Figures 1, 2A, 4A). These defects are speculated to be caused by cellular stresses generated under this control condition, whereas the wild-type strain might have adapted to the stresses through a basal level of autophagy. The high ROS levels in the *Cgatg1*Δ strain in the absence of any exogenous ROS agents are considered to be caused by the defect in metabolism of endogenous ROS generated by *C. glabrata* basal activities such as respiration. The endogenous ROS may also be a cause of the slow growth of *Cgatg1*Δ strain. Indeed, the doubling time of *Cgatg1*Δ strain was shortened to some extent by *CTA1* overexpression, although it was not rescued to the level of wild-type strain (Figures 4D,E). Stimuli other than ROS such as excessive ions, high osmolarity, and high temperature were analyzed, but the *Cgatg1*Δ strain was not sensitive to these stresses (Supplementary Figure S3).

The function of autophagy in the adaptation to starvation is supposed to enhance *C. glabrata* viability in hosts, as nutrient conditions are thought to be unstable in various host environments. In addition, the effect of autophagy on ROS metabolism and resistance to H_2_O_2_ in *C. glabrata* was predicted to contribute to its survival against ROS-generating immune defenses, such as macrophages. An *ex vivo* experiment using mouse peritoneal macrophages revealed low viability of the *Cgatg1*Δ strain in the macrophages (Figure 5). This defect can be attributed to the decreased resistance of the *Cgatg1*Δ strain to both nutrient starvation and to the H_2_O_2_ encountered when phagocytosed by macrophages.

*In vivo* mouse experiments confirmed that autophagy is a ‘fitness factor’ in *C. glabrata*. Differences between the wild-type strain and the *Cgatg1*Δ strain are thought to originate from the basic growth rate, the resistance to insufficient nutrient environments, and the oxidative stress response to macrophages, as well as other effects of autophagy that were not addressed in this study. Both the IAC mouse model, which was employed to examine effect of peritoneal macrophages, and the DC mouse model showed attenuated viability of the *Cgatg1*Δ strain. These results suggest that immune defense not only by macrophages but also by other monocytes in the blood and subsequently infected organs might contribute to the decreased CFU of the *Cgatg1*Δ strain. However, the number of CFU from kidneys was not significantly different between the wild-type strain and the *Cgatg1*Δ strain in the DC mouse model. Although the exact reason is unclear, one possible explanation is that renal macrophages concentrate to the tubulointerstitium (Xu and Shinohara, 2017), a compartment of the kidney, and might not respond effectively to *C. glabrata* cells that usually invade the vasculature and nephrons in the kidney. Renal macrophages have a role in host defense against *C. albicans* (Lionakis et al., 2013; Ngo et al., 2014), but this function may be dependent on the *Candida* species. *C. glabrata* infection does not strongly attract immune cells, including neutrophils, and thus rarely causes severe inflammation (Kasper et al., 2015). Nevertheless, the results suggest that the extent of influence of autophagy in *C. glabrata* is substantially different among infected organs and tissues.

Although our experiments *in vitro* and *ex vivo* suggested the possibility of its contribution to virulence, obvious differences in viability and health status were not observed between the mice infected with the wild-type and *Cgatg1*Δ strains. From our present results *in vivo, C. glabrata* autophagy is concluded to be a fitness factor for longer survival in hosts.

## Conclusion

Atg1 has been broadly investigated in many eukaryotes and established as a key player in autophagy induction (Wang and Kundu, 2017). However, reports of autophagy and Atg1 focusing on virulence are limited, especially in *Candida* species. The current study demonstrated for the first time that Atg1 is required for autophagy in *C. glabrata* (Graphical Abstract). Persistence in hosts is a characteristic of *C. glabrata* infection. The *Cgatg1*Δ phenotypes indicate that autophagy may play an important role in fitness and survival of this fungus in infected hosts. The detailed characteristics of CgAtg1, including functional mechanisms, modifications, and interactions with other factors, need to be elucidated in future studies.

## Author Contributions

SS and TM designed the project, conducted the experiments, analyzed the data, and prepared the manuscript. All authors reviewed and approved the final version of the manuscript.

## Conflict of Interest Statement

The authors declare that the research was conducted in the absence of any commercial or financial relationships that could be construed as a potential conflict of interest.

## Footnotes

1 http://www.candidagenome.org

## Abbreviations

ATG: autophagy-related
CFU: colony forming units
Cg: *Candida glabrata*
*CTA1*: catalase A 1
DC: disseminated candidiasis
DCF: dichlorofluorescein
DMEM: Dulbecco’s modified Eagle’s medium
ECL: enhanced chemiluminescence
FBS: fetal bovine serum
GFP: green fluorescent protein
H_2_O_2_: hydrogen peroxide
IAC: intra-abdominal candidiasis
ORF: open reading frame
PBS: phosphate-buffered saline
qRT-PCR: quantitative reverse transcription-polymerase chain reaction
ROS: reactive oxygen species
SC-trp: synthetic complete medium lacking tryptophan
SD-N: synthetic dextrose medium lacking nitrogen
SE: standard error
YPD: yeast extract peptone dextrose
