# Supplementary figures and images for: Autophagy-Inducing Factor Atg1 Is Required for Virulence in the Pathogenic Fungus Candida glabrata
Source: Front Microbiol. 2019 Jan 25;10:27. doi: 10.3389/fmicb.2019.00027 (PMC6362428; doi:10.3389/fmicb.2019.00027)

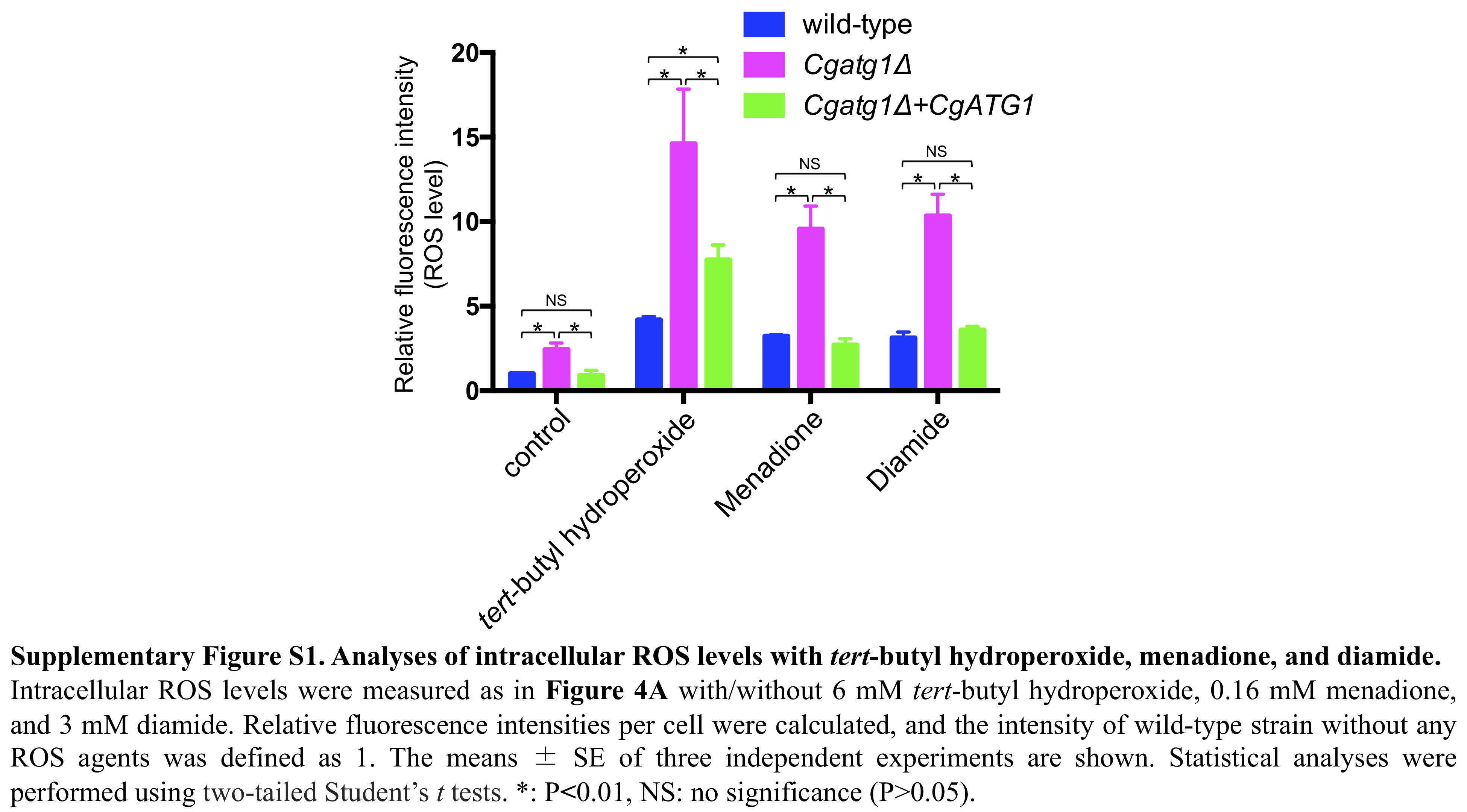

Supplement: Supplementary file 1 [file Image_1.jpg]

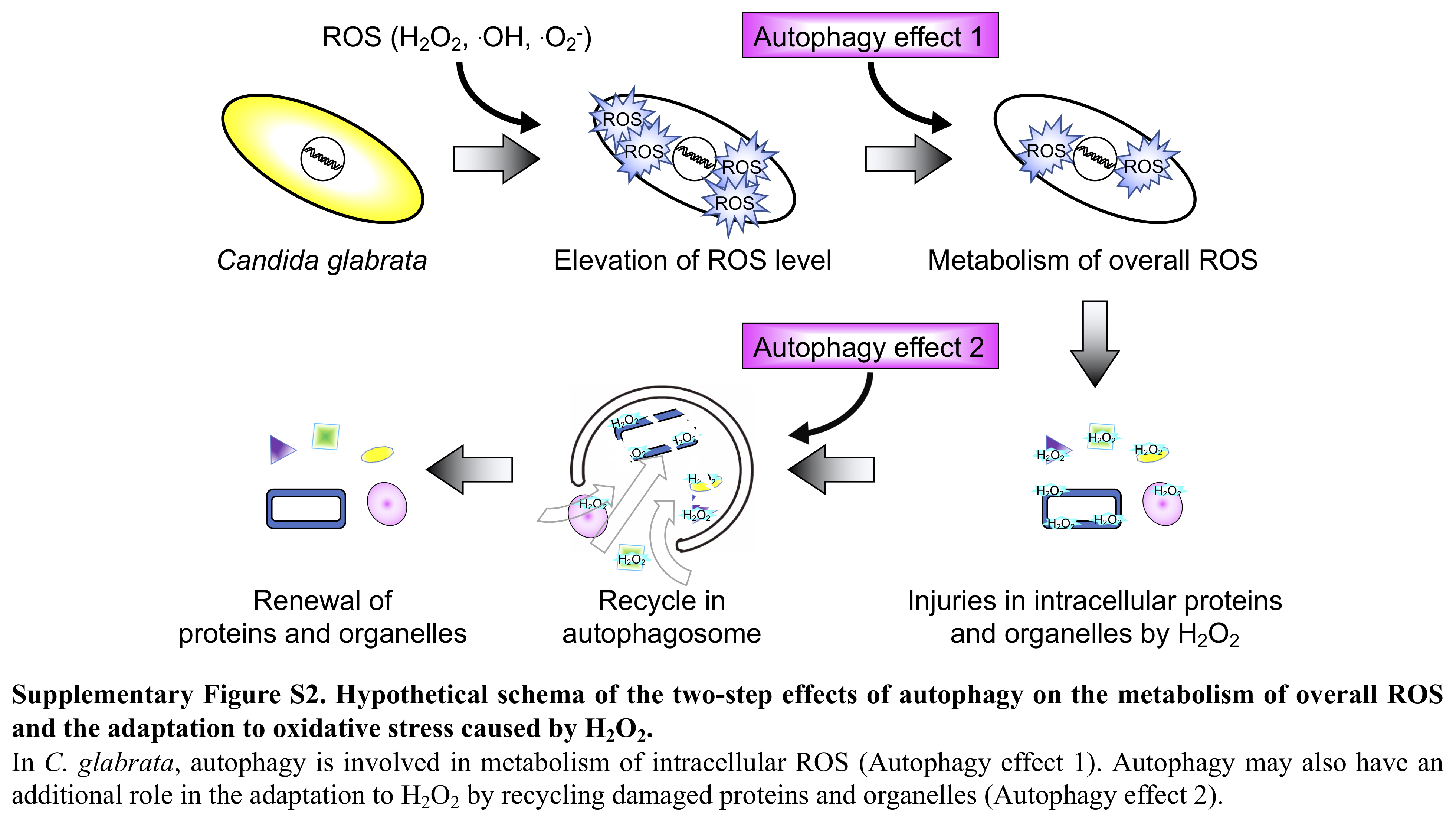

Supplement: Supplementary file 2 [file Image_2.jpg]

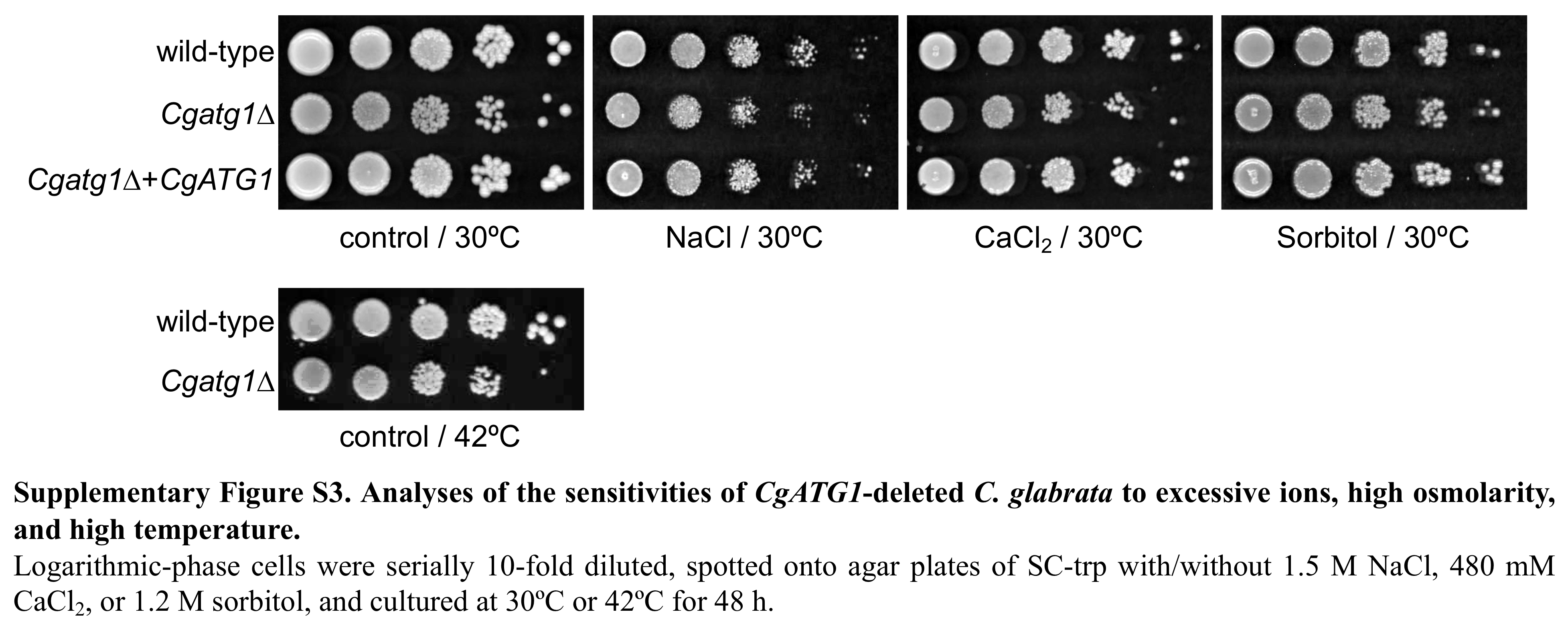

Supplement: Supplementary file 3 [file Image_3.jpg]
